# Reporting of health equity considerations in equity-relevant observational studies: Protocol for a systematic assessment

**DOI:** 10.12688/f1000research.122185.1

**Published:** 2022-06-06

**Authors:** Omar Dewidar, Tamara Rader, Hugh Waddington, Stuart G Nicholls, Julian Little, Billie-Jo Hardy, Tanya Horsley, Taryn Young, Luis Gabriel Cuervo, Melissa K Sharp, Catherine Chamberlain, Beverley Shea, Peter Craig, Daeria O Lawson, Anita Rizvi, Charles Shey Wiysonge, Tamara Kredo, Miriam Nkangu Nguliefem, Elizabeth Ghogomu, Damian Francis, Elizabeth Kristjansson, Zulfiqar Bhutta, Alba Antequera Martin, G J Melendez-Torres, Tomas Pantoja, Xiaoqin Wang, Janet Jull, Janet Hatcher Roberts, Sarah Funnell, Howard White, Alison Krentel, Michael Johnson Mahande, Jacqueline Ramke, George A Wells, Jennifer Petkovic, Peter Tugwell, Kevin Pottie, Lawrence Mbuagbaw, Vivian Welch

**Affiliations:** 1 Bruyère Research Institute, University of Ottawa, Ottawa, Canada; 2 Freelance health research librarian, Ottawa, Canada; 3 London School of Hygiene and Tropical Medicine, London, UK; 4 Clinical Epidemiology Program, Ottawa Hospital Research Institute, Ottawa, Canada; 5 School of Epidemiology and Public Health, University of Ottawa, Ottawa, Canada; 6 Waakebiness Bryce Institute for Indigenous Health, Dalla Lana School of Public Health, Toronto, Canada; 7 Royal College of Physicians and Surgeons of Canada, Ottawa, Canada; 8 Centre for Evidence Based Health Care, Department of Global Health, Stellenbosch University, Stellenbosch, South Africa; 9 Unit of Health Services and Access, Department of Health Systems and Services, Pan American Health Organization, Washington, DC, USA; 10 Health Research Board Centre for Primary Care Research, Department of General Practice, Royal College of Surgeons in Ireland, Dublin, Ireland; 11 Judith Lumley Centre, School of Nursing and Midwifery, La Trobe University, Melbourne, Australia; 12 Ngangk Yira Research Centre for Aboriginal Health and Social Equity, Murdoch University, Perth, Australia; 13 Institute of Health and Wellbeing, University of Glasgow, Glasgow, UK; 14 Department of Health Research Methods, Evidence, and Impact, McMaster University, Hamilton, Canada; 15 School of Psychology, University of Ottawa, Ottawa, Canada; 16 Cochrane South Africa, South African Medical Research Council, Cape Town, South Africa; 17 School of Health and Human Performance, Georgia College, Milledgville, USA; 18 Centre for Global Child Health, Hospital for Sick Children, Toronto, Canada; 19 Institute for Global Health & Development, The Aga Khan University, Karachi, Pakistan; 20 Biomedical Research Institute Sant Pau, Hospital de la Santa Creu i Sant Pau, Barcelona, Spain; 21 University of Exeter College of Medicine and Health, Exeter, UK; 22 Family Medicine Department, School of Medicine, Pontifcia Universidad Catolica de Chile, Santiago, Chile; 23 Michael G. DeGroote Institute for Pain Research and Care, McMaster University, Hamilton, Canada; 24 Faculty of Health Sciences, School of Rehabilitation Therapy, Queen’s University, Kingston, Canada; 25 WHO Collaborating Centre for Knowledge Translation and Health Technology Assessment in Health Equity, Ottawa, Canada; 26 Department of Family Medicine, Queen’s University, Kingston, Canada; 27 Department of Family Medicine, Faculty of Medicine, University of Ottawa, Ottawa, Canada; 28 Campbell Collaboration, Oslow, Norway; 29 Department of Epidemiology & Biostatistics, Institute of Public Health, Kilimanjaro Christian Medical College, Moshi, Tanzania; 30 International Centre for Eye Health, London School of Hygiene & Tropical Medicine, London, UK; 31 School of Optometry and Vision Science, University of Auckland, Auckland, New Zealand; 32 University of Ottawa Heart Institute, Ottawa, Canada; 33 Faculty of Medicine, School of Epidemiology and Public Health, University of Ottawa, Ottawa, Canada; 34 C.T. Lamont Primary Care Research Centre, Bruyère Research Institute, Ottawa, Canada; 35 Department of Family Medicine, Schulich school of Medicine & Dentistry, Western University, London, Canada

**Keywords:** Observational studies, health equity, research reporting, research methodology, study design

## Abstract

**Background:** The mitigation of unfair and avoidable differences in health is an increasing global priority. Observational studies including cohort, cross-sectional and case-control studies tend to report social determinants of health which could inform evidence syntheses on health equity and social justice. However, the extent of reporting and analysis of equity in equity-relevant observational studies is unknown.

**Methods:** We define studies which report outcomes for populations at risk of experiencing inequities as “equity-relevant”. Using a random sampling technique we will identify 320 equity-relevant observational studies published between 1 January 2020 to 27 April 2022 by searching the MEDLINE database. We will stratify sampling by 1) studies in high-income countries (HIC) and low- and middle-income countries (LMIC) according to the World Bank classification, 2) studies focused on COVID and those which are not, 3) studies focused on populations at risk of experiencing inequities and those on general populations that stratify their analyses. We will use the PROGRESS framework which stands for place of residence, race or ethnicity, occupation, gender or sex, religion, education, socioeconomic status, social capital, to identify dimensions where inequities may exist. Using a previously developed data extraction form we will pilot-test on eligible studies and revise as applicable.

**Conclusions:** The proposed methodological assessment of reporting will allow us to systematically understand the current reporting and analysis practices for health equity in observational studies. The findings of this study will help inform the development of the equity extension for the STROBE (Strengthening the Reporting of Observational studies in Epidemiology) reporting guidelines.

## Introduction

Less than a decade remains until the 2030 deadline set by the United Nations to meet the 17 Sustainable Development Goals ^1^ aimed at global economic, social, and environmental transformation. One of the 17 goals, and a cross-cutting theme across several others, ^2^ is health equity, defined as the absence of differences in health that are avoidable and unjust. ^3^ The achievement of health equity as a priority has been widely endorsed by several international organizations ^4^^,^^5^ and resonates with the moral imperative of social justice and recognition of human rights principles.

A major challenge is the lack of evidence that includes thoughtful, rigorous collection of health equity data to inform program development and policymaking. ^6^ In fact, global organizations such as the World Health Organization (WHO) and the Pan American Health Organization (PAHO) have called for the improvement of equity considerations in the reporting of research. ^7^^–^^10^ Thus, the integration of health equity in research is needed in both primary and secondary research. ^11^^–^^14^ In primary research, observational studies are particularly well-suited for investigating health equity as they often include populations at risk of experiencing inequities. ^15^^,^^16^ Observational studies are analytical or descriptive studies evaluating a research question without changing exposure to an intervention. ^17^ In the absence of a control group, they can be descriptive in nature but do not permit the investigation of causal associations. ^18^ Observational studies predominate in health-related research, ^19^^,^^20^ and are used to investigate numerous types of research questions, such as detecting rare or late adverse effects of treatments and to explore complex systems. ^21^ Furthermore, observational studies have highlighted the vast inequities in society during the COVID-19 pandemic, ^22^ and simultaneously, have had an important role in informing public health responses. ^23^^,^^24^

The well-known STROBE (Strengthening Reporting of Observational studies in Epidemiology) reporting guideline, which is intended to be used for the reporting of observational studies, is used by over 60% of authors of observational studies. ^25^ However, despite widespread use of the STROBE reporting guidelines, there is a persistent lack of integration and reporting of sex and gender (among other dimensions) in observational studies. ^26^^–^^28^ Comprehensive reporting in research is essential to assess its reliability, reproducibility and methodological rigour, and ensure that the research meets the needs of the potential users. ^29^ Using reporting guidelines contributes to meeting these goals by increasing the completeness and transparency of research papers. ^30^^–^^33^ The inadequate reporting may be, in part, due to lack of guidance on reporting equity items in such studies.

Complex health systems questions and addressing social determinants of health may require multi-stakeholder and intersectoral collaboration and engagement, for which equity perspectives can serve as a linchpin. ^34^ Observational studies also allow for addressing questions on health policy, health systems, and health services organization, delivery, prioritization, and implementation. ^35^^–^^37^ Different frameworks are available to describe factors associated with health equity. ^38^^,^^39^ The Campbell and Cochrane Equity Methods Group, for example, proposes using the PROGRESS-Plus framework. This acronym is widely used and stands for Place of residence, Race or ethnicity, Occupation, Gender or sex, Religion, Education, Socioeconomic status, and Social capital. ^39^ The “plus” accounts for additional characteristics that could result in inequitable health outcomes. Although the reporting on equity in observational research would increase the value of research outcomes through the recognition of how these dimensions impact interventions, the application and use of equity frameworks in reporting health research are not yet standard practice. Therefore, our objective is to systematically evaluate how equity-relevant observational studies describe characteristics of their samples, design features and analysis, and interpretation of their findings across PROGRESS-Plus; we also aim to use the findings of this study to inform the development of an equity reporting guideline.

## Methods

**Figure 1.**
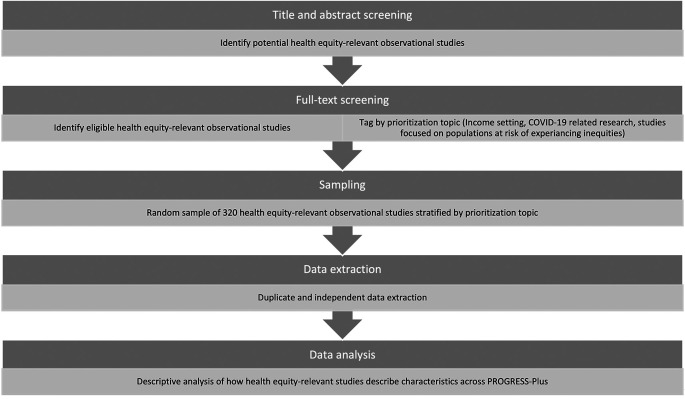
Study flow diagram.

Figure 1 outlines the overarching process to be followed in the conduct of this study; from identifying relevant studies at title and abstract stage to data extraction and analysis.

### Eligibility criteria

We will include health equity-relevant observational studies from all settings, published between 1 January 2020, and 27 April 2022. We define health equity, health equity-relevant studies and observational studies in Table 1. We chose to focus on equity-relevant studies, since these are most likely to have methods of describing populations experiencing inequities, and are most likely to include methods to assess similarities or differences in effects for populations experiencing inequities.

**Table 1.** Definitions of terms used in the methodological assessment.

Term	Definition and explanation
Health inequity	Health inequity is defined as avoidable and unfair differences in health. ^3^
Health equity-relevant studies	We define “health equity-relevant” as studies that focus on individuals or populations experiencing inequities or studies of mixed populations that analyzed at least one health outcome across one or more PROGRESS factor. ^12^^,^^40^ We define health outcomes according to the WHO definition, as “changes as in health status that result from the provision of health services”. ^41^ This includes studies focused on populations at risk of experiencing inequities such as people who are homeless and asylum seekers, as well as studies which present disaggregated results or evaluate outcomes across one or more PROGRESS factors. However, studies which control for PROGRESS in analyses without analyzing differences across the factors are not considered equity-relevant. For example, studies of breast cancer in women would not be considered health equity-relevant since women are not disadvantaged in opportunities for health as it relates to breast cancer care. However, if stratified analysis was done to compare mortality of women living in rural vs urban area (or other PROGRESS factors), the study would be considered equity-relevant. Similarly, a study of low-income or racialized women accessing breast cancer care would be considered health equity-relevant because they experience different opportunities in obtaining care.
Observational studies	We define observational studies as analytical or descriptive studies evaluating a research question without changing exposure to an intervention. ^17^^,^^18^ Observational studies are classified in the STROBE reporting guideline into three types: 1) cohort: following an exposed population over time; 2) case-control: comparing exposures between people with a particular disease outcome (cases) and people without that outcome (controls); and 3) cross sectional: assessing all individuals in a sample at the same point in time. ^21^ Studies conducted using routinely collected data stored in administrative datasets can be classified into these three types of studies. ^42^

We will not include studies focused on Indigenous populations in this evaluation, as they will be assessed in a separate but complementary study led by Indigenous researchers as it will require a different sampling approach and methodological assessment. We decided not to use “Plus” criteria for eligibility since they require a greater deal of judgement regarding the contextual factors that may result in inequities. We will exclude case series and case reports since they do not include a reference population or comparison group enabling assessment of differences across PROGRESS factors.

### Sampling strategy

Based on consultation with our interdisciplinary team of researchers and decision-makers, and patients and public, we will use a three-factor, randomized sampling strategy to balance: 1) studies in high-income countries (HIC) and low- and middle-income countries (LMIC) according to the World Bank classification, 2) studies focused on COVID-19 and those which are not, 3) studies focused on populations experiencing inequities and those that stratify their analyses. We chose to stratify by country because of the importance of the context and resources in LMIC settings which affect health inequities both among LMIC and between LMIC and HIC, ^43^^–^^45^ Given the evidence on exacerbation of inequities during the COVID-19 pandemic, we decided to ensure balance across studies related to COVID-19 and those that are not. ^46^

Although we chose only three factors for the sampling strategy, we will assess all aspects of PROGRESS-Plus in the included studies; thus other populations experiencing inequities will be included, such as people who are living with low income, homeless, migrants, asylum-seekers, and racialized individuals. Table 2 lists examples of studies that meet our eligibility criteria.

**Table 2.** Examples of health equity-relevant observational studies published after 2020.

	LMIC		HIC	
	COVID+	COVID-	COVID+	COVID-
General population- *i.e.* mixed	Epidemiological characteristics of patients with severe COVID-19 infection in Wuhan, China: evidence from a retrospective observational study ^47^	Effectiveness of seasonal malaria chemoprevention at scale in west and central Africa: an observational study ^48^	Patterns of use of secondary mental health services before and during COVID-19 lockdown: observational study ^49^	School Entry, Educational Attainment, and Quarter of Birth: A Cautionary Tale of a Local Average Treatment Effect ^50^
Focused on populations experiencing inequities	Ethnic and regional variations in hospital mortality from COVID-19 in Brazil: a cross-sectional observational study ^51^	Demystifying the factors associated with rural–urban gaps in severe acute malnutrition among under-five children in low- and middle-income countries: a decomposition analysis ^52^	Hesitant or Not? The Association of Age, Gender, and Education with Potential Acceptance of a COVID-19 Vaccine: A Country-level Analysis ^53^ A case-control and cohort study to determine the relationship between ethnic background and severe COVID-19 ^54^	Health Care Utilization Before and After the “Muslim Ban” Executive Order Among People Born in Muslim-Majority Countries and Living in the US ^55^

### Sample size

We will randomly select a sample of 320 equity-relevant observational studies, since assessing all potential eligible studies would be unmanageable for our review team. To ensure we assess a sample that provides comparable results, we conducted sample size calculation for binary outcomes (categorical questions for consideration of equity in the studies) with 95% confidence intervals of ± 6% for observed proportions of 50% ( *i.e.* assuming that half of the studies would report at least one PROGRESS characteristic). This sample size has also been used in similar studies. ^56^^–^^59^ We acknowledge that the study does not have sufficient power to allow a comparison of reporting across the three prioritized groups, so we will only report data descriptively. Eligible studies will be exported into Microsoft Excel and sorted using a random sequence using the built-in random-number generator. We will sample the studies as specified in Table 3.

**Table 3.** Sampling strategy for equity-relevant studies across the prioritized topics.

	LMIC		HIC		Total
	COVID+	COVID-	COVID+	COVID-	
General population	40	40	40	40	**160**
Focused on populations experiencing inequities	40	40	40	40	**160**
Total	**80**	**80**	**80**	**80**	**320**

COVID+: studies related to COVID-19, COVID-: studies not related to COVID-19. LMIC: Low-middle income countries, HIC: High Income countries.

### Search strategy

We have designed a search strategy in collaboration with a librarian (TR) experienced in systematic reviews, using a method designed to optimize term selection. ^60^ A validated search filter to identify equity-focused studies was adapted for use in this study, ^61^ and additional terms to increase retrieval of papers about indigenous populations were added (lines 213-232). ^62^^,^^63^ These were developed in collaboration with our Indigenous Steering Committee. Studies related to Indigenous populations will be identified from these search terms and will be utilized to conduct an independent but highly complementary study focused on Indigenous populations.

We used a validated search filter for observational studies with two extra terms to retrieve cross-sectional studies. ^64^ We further added the terms “Instrumental variable”, “discontinuity design”, “interrupted time series”, “discontinuity design”, “matching”, “synthetic control” and “difference-in-difference” to our search strategy to ensure that we capture observational econometric studies that meet our eligibility criteria. Review articles and randomized controlled trials will be excluded from these results using validated study design filters for Ovid MEDLINE. ^65^ The draft search strategy is stored in the Open Science Framework (OSF) repository ( *Extended data* ^67^). We will test the search strategy with a set of 10 studies that we identify to be eligible.

We will restrict our search to the last two years because we are interested in describing current analysis and reporting of equity, not trend over time. This period also captures the girth of the literature published throughout the pandemic. We conducted a preliminary search of Ovid MEDLINE for papers published between 1 January 2020, to 17 April 2022 and obtained 16,263 citations. Due to the high volume of results retrieved, we decided that searching only one database would be feasible. In the conduct of this study, we expect an adequate number of observational studies in this period to meet our eligibility criteria.

### Screening

Studies will be screened at title and abstract level by a single investigator using DistillerSR software. ^66^ One investigator will screen the full texts of studies, and OD will validate. Conflicts will be resolved through discussion with a third reviewer at weekly meetings. We will use the DistillerSR randomization function to randomly sample citations at a set interval of 20% of unreviewed citations until we reach the planned sample size.

At full-text screening, we will tag eligible studies according to our prioritized topics (Type of equity relevance, country income setting, COVID-19 research).

### Data extraction

Data will be extracted independently by two extractors. We will develop and pretest a data extraction form using DistillerSR. We will conduct training on the data collection with data extractors using sets of three to five studies at a time, until there is 80% agreement across all items between extractors. Conflicts will be resolved by discussion between extractors and reaching consensus. We will develop a data dictionary to ensure consistent extraction across team members and refine during pre-testing.

We will capture study characteristics including the purpose of the study (focused on populations experiencing inequities or whether the study included general populations in which there may be populations experiencing inequities), title/abstract, background/rationale, study design, population characteristics, results, analyses, interpretation of applicability and discussion. We will collect details on the motivation behind studies for a separate evaluation of the way equity is defined in these studies and the motivation for studying equity-seeking populations. The complete list of items is provided in our OSF repository ( *Extended* data ^67^).

We will assemble the author contact details for a separate survey of authors (of the included studies) to ask about engagement with populations experiencing inequities, since we believe that engagement with populations experiencing inequities informs better reporting on health equity. The reason for conducting the survey is because we expect that some of the results related to equity would not be well reported in the articles.

### Data analysis

We will descriptively present the reporting of each item for the observational studies in our sample by tabulating the data for each item to assess the frequency of reporting. We will conduct subgroup analysis on studies that considered health equity as a reason for conducting the study.

### Dissemination

Our findings will be shared on pre-print servers and submitted for peer-review publication. In addition, we plan on sharing our findings on Twitter and at relevant conferences for wider dissemination.

## Discussion

The primary objective of this study will be to describe the reporting and analysis of equity in equity-relevant observational studies. By focusing on studies that meet this criteria, we will document studies that have missed opportunities for providing information regarding populations at risk of experiencing inequities. Moreover, this exercise may help us identify examples of equity considerations in observational studies, according to our candidate items for the equity extension for STROBE reporting guideline, to illustrate how and to what extent they are presented in the literature.

Our study is not without limitations. First, our search of only MEDLINE would be expected to yield studies mostly from HIC and focused on clinical topics. However, we plan to mitigate this selection bias by purposefully stratifying sampling across country income setting and randomly selecting a sample from the eligible studies. Second, although a third of early COVID-19 literature has been shared as preprints, we will not include studies from pre-print servers as studies in peer-review journal articles tend to show substantial improvements in the quality of reporting compared to pre-prints. Therefore, it may not be appropriate to pool preprints with peer-reviewed published articles. Future work could aim to investigate the difference in reporting of health equity in articles published in pre-print servers compared to peer-reviewed articles.

The proposed list of extraction items is extensive and provides several gateways for future projects to fill gaps in understanding the integration and reporting of equity in research. Using the results of this methodological assessment, we plan to conduct a sociological discourse analysis of motivation for assessing health equity in equity-relevant observational studies. In accordance with the plan for this extension, we will conduct an independent study on Indigenous research, led by Indigenous researchers, to identify and describe well-reported studies using the CONSIDER checklist. Furthermore, the selective sampling across prioritized groups provides an opportunity to conduct more focused investigations, tailored to each respective topic ( *i.e.* LMIC, COVID-19 research).

## Data availability

### Underlying data

No data are associated with this protocol.

### Extended data

Open Science Framework: STROBE-equity reporting guidelines, https://osf.io/cp3z2. ^67^

This project includes the following extended data:

• Search strategy.docx
• Extraction items mapped to the STROBE-Equity candidate items.docx

Data are available under the terms of the Creative Commons Attribution 4.0 International license (CC-BY 4.0).
