# Supplementary material for: Reporting of health equity considerations in equity-relevant observational studies: Protocol for a systematic assessment
Source: F1000Res. 2022 Jun 6;11:615. [Version 1] doi: 10.12688/f1000research.122185.1 (PMC12413612; doi:10.12688/f1000research.122185.1)
Supplement: Peer review history

# Reviewer report: Reviewer response for version 1

**DOI:** 10.5256/f1000research.134140.r171061

**Published:** 2025-09-05

**Authors:** Mireya Martinez Garcia

**Affiliations:** 1 Sociomedical Research, National Institute of Cardiology "Ignacio Chávez", Mexico City, Mexico

I read with interest the paper entitled “Reporting of health equity considerations in equity-relevant observational studies: Protocol for a systematic assessment”, the idea is original and the study's findings will surely contribute to inform the development of an equity reporting guide.

1. **Introduction:** The authors could mention some examples of the inequities highlighted by the observational studies during the COVID-19 pandemic, and their impact in informing public health responses.
  The authors mention that “Complex health systems questions and addressing social determinants of health may require multi-stakeholder and intersectoral collaboration and engagement, for which equity perspectives can serve as a linchpin” Could you be more specific and mention a few examples briefly?
  The authors mention that the “objective is to systematically evaluate how equity relevant observational studies describe characteristics of their samples, design features and analysis, and interpretation of their findings across PROGRESS-Plus” also in the “Figure 1. Study flow diagram” or in Table 2 are mentioned, but in the Methods/Eligibility criteria you decided not to use “Plus” criteria for eligibility since they require a greater deal of judgment regarding the contextual factors that may result in inequities … the authors could clarify it.
2. **Methods:** In the Methods/Eligibility criteria section you mentioned “We will not include studies focused on Indigenous populations… it will require a different sampling approach and methodological assessment.” but in the Search strategy then you said “…and additional terms to increase retrieval of papers about indigenous populations were added”. The way of presenting the text is a bit confusing, although it is understood that a complementary study will be carried out in collaboration with the Indigenous Steering Committee. How did it affect the final count of the studies recovered by including those related to the indigenous population?
  Although the authors include the reference (Bramer WM, de Jonge GB, Rethlefsen ML, et al.), please briefly describe the method designed to optimize term selection mentioned in the Search strategy section.
  Why do the authors believe that using the MEDLINE database is sufficient for their search? beyond the high volume of results obtained in the preliminary search. What advantages or disadvantages does this recovery system have? 
  Please briefly describe the DistillerSR software and why that was your favorite for the screening step?
  The complete list of items regarding motivation behind studies evaluation is in an OSF repository. Could the authors give some examples or more details of them? to facilitate the reader a more fluent reading of the protocol and not have to consult supplementary material or the Extended data each time.

Thanks

Is the study design appropriate for the research question?

Yes

Is the rationale for, and objectives of, the study clearly described?

Yes

Are sufficient details of the methods provided to allow replication by others?

Yes

Are the datasets clearly presented in a useable and accessible format?

Yes

Reviewer Expertise:

Health Systems; Health Policy; Social Analysis of Healthcare; Socio-medical Research

I confirm that I have read this submission and believe that I have an appropriate level of expertise to confirm that it is of an acceptable scientific standard.

# Reviewer report: Reviewer response for version 1

**DOI:** 10.5256/f1000research.134140.r183191

**Published:** 2023-08-17

**Authors:** Ihoghosa Iyamu

**Affiliations:** 1 The University of British Columbia, Vancouver, British Columbia, Canada

Thanks for the opportunity to review this study protocol. It focuses on an important topic of health equity analyses and reporting in scientifica articles and this is very important. There are however opportunities to provide additional detail that can improve the quality and replicability of the study.

 My comments are outlined as major and minor comments below:

 Major comments 

1. There appears to be a struggle to balance feasibility with rigour and the idea of a comprehensive study. For example, while the authors note their intent to search only one database as a limitation, I wonder if it makes sense to have a more specific question that allows the team search more databases to ensure a less biased sampling frame for the study. The Cochrane group recommends searching Embase and Medline at least, in addition to other registries. https://training.cochrane.org/handbook/current/chapter-04
  If the questions are more specific, that might be achieved.
2. More specific details are needed for the screening procedures. Are the authors screening all the 16,000 plus titles and abstracts returned in the search, is there some automation going into the screening process especially because the authors use DistillerSR? Further, some studies do stratified analyses of outcomes as sub-analyses but may not include this in the abstract. Are there chances that your current approach and framing of the questions may miss these sort of studies? What is the protocol to ensure that papers of interest are not inadvertently excluded due to the current strategy. On the random sampling, it would also be great to know if this is a sampling with replacement or not.
3. The authors describe a follow-up survey as part of this study but only do so in passing. Questions about the survey include; What kinds of questions will you be asking? Is it a simple "did you engage with populations experiencing inequities?", How do you define engagement? What will be your threshold for saying engagement was done or not done? More information is required for clarity on this.
4. One also wonders why only a 2 year window was chosen for the study. One wonders if the last 2 years appropriately capture scientific practices and protocols or if this period is greatly biased by COVID and not necessarily reflective of the spectrum of practices within the scientific community. For example, were engagements with historically marginalized communities not altered during the pandemic? How is that reflected in the studies included?
5. One is not sure about the framework being adhered to in this study. For example, is there a PRISMA guideline or something similar that this largely follows?

Minor comments 

1. The first instance of "equity-relevant" studies should be accompanied by a reference to the definition. This was only done later and could be confusing.
2. What is the justification for 80% agreement? What would that amount to in terms of say a Cohen's Kappa or some other standardized form of agreement metrics?
3. Authors state they will only use the PROGRESS framework, but in sections of the manuscript, they refer to PROGRESS-Plus

Is the study design appropriate for the research question?

Yes

Is the rationale for, and objectives of, the study clearly described?

Yes

Are sufficient details of the methods provided to allow replication by others?

Partly

Are the datasets clearly presented in a useable and accessible format?

Not applicable

Reviewer Expertise:

Health services research; Digital health equity; sexual health

I confirm that I have read this submission and believe that I have an appropriate level of expertise to confirm that it is of an acceptable scientific standard, however I have significant reservations, as outlined above.

# Reviewer report: Reviewer response for version 1

**DOI:** 10.5256/f1000research.134140.r151179

**Published:** 2022-12-16

**Authors:** Khic-Houy Prang

**Affiliations:** 1 University of Melbourne, Melbourne, Australia

Thank you for the opportunity to read your study protocol which aims to develop a health equity reporting guideline for observational studies. I commend the authors on undertaking an ambitious review across diverse settings and population groups to identify health equity relevant items. Further clarifications about the health equity definition, inclusion and exclusion criteria of the review are required.

1. I agree that health equity is important to capture in observational studies, however health equity is rather a difficult construct to measure that embody many social determinants and individual factors that influence access to care and ultimately health outcomes. I am unclear how you will differentiate between health equity studies and non-health equity studies. Most (if not all) observational studies will generally include a demographic table of their sample, as such based on your inclusion criteria then all studies would be considered a health equity-relevant study? I am not sure whether sub-group analysis will be enough to identify health equity studies as these studies may reflect health disparity? How will you differentiate between health equity and health disparity studies? Perhaps it might help to narrow the focus to intersectionality analysis?
2. The definition of population at risk of experiencing inequities is broad and includes all type of population. It would be difficult to identify as this is likely to be dependent on the disease and the context, and the comparison group. Will all the LMICs studies automatically be considered a population at risk because of the setting? What about specific population groups in HICs? You gave some examples of at-risk groups such as migrants and asylum seekers. Will it be worth considering at the outset who these groups are and narrow the scope of the review?
3. Can you clarify why you chose PROGRESS Plus over other health equity frameworks to guide the review?
4. The inclusion criteria are observational studies and observational econometric studies. What about quasi-experimental and natural experiment studies given the focus on COVID studies?
5. The proposed review includes a systematic search approach but does not include any quality assessment or risk of bias. Could you please clarify what type of review this is?
6. Is there a chance that you may missed studies using only one search engine? What about the grey literature? Will limiting the time period to two years (2020-2022) skew your search to predominantly COVID studies given the prioritisation of COVID studies published during this period over non-COVID studies?
7. Given that your research question is rather broad and does not focus on a specific population groups, interventions or health outcomes, the search strategy is rather complex. It seems to be based on the factors in the PROGRESS framework, I wonder if it would be useful to limit the search to observational study designs and heath equity-related terms, although noting that studies without using health equity-related terms will unlikely be picked up in the search. Given that the focus of the study is on health equity specifically, then perhaps it may be better to focus on such studies and describe what these studies have reported? Best practice for systematic and scoping reviews involves two investigators screening the full text articles.
8. There are almost 40 authors listed in the paper. This seems excessive for a protocol paper, with most of the authors’ contribution limited to reviewing and editing. Do all members in each of the committees warrants authorship?
9. Minor edits: A typo in figure 1 ‘experiencing inequities’. In tables 2 and 3, it would be helpful perhaps to not use ‘COVID +’ or ‘COVID –‘ as the headers as it appeared to denote COVID studies with positive or negative results, perhaps COVID-related studies and non-COVID related studies may be more suitable? Reference required on page 7 regarding the survey - ‘ […] that engagement with populations experiencing inequities inform better reporting on health equity’.

Is the study design appropriate for the research question?

Partly

Is the rationale for, and objectives of, the study clearly described?

Yes

Are sufficient details of the methods provided to allow replication by others?

Partly

Are the datasets clearly presented in a useable and accessible format?

Not applicable

Reviewer Expertise:

Health services and systems research, evaluation and implementation science, mixed-methods research

I confirm that I have read this submission and believe that I have an appropriate level of expertise to confirm that it is of an acceptable scientific standard, however I have significant reservations, as outlined above.
